# Solvent-Dependent Chemical Profiles and Biological Activities of *Pueraria lobata* Root Extracts

**DOI:** 10.3390/molecules31060965

**Published:** 2026-03-13

**Authors:** Ji-Hyun Lee, Ji-Ye Lim, Dae-Ki Kim, Dae-Ho Yun, Yong-Deok Jeon, Dong-Hyun Lee

**Affiliations:** 1 Department of Korean Pharmacy, Woosuk University, Wanju-Gun 55338, Republic of Korea; jihyunsh1211@naver.com; 2 Department of Immunology, Institute of Medical Sciences, Medical School, Jeonbuk National University, Jeonju 54907, Republic of Korea; 84juce@naver.com (J.-Y.L.); daekim@jbnu.ac.kr (D.-K.K.); 3 Department of Health and Medical Administration, Baewha Women’s University, Seoul 03039, Republic of Korea; ydh016@hanmail.net; 4 Department of Obstetrics and Gynecology, Jeonbuk National University Hospital, Jeonju 54907, Republic of Korea

**Keywords:** *Pueraria lobata*, extraction solvent, phytochemical profiling, antioxidant activity, anti-inflammatory activity

## Abstract

*Pueraria lobata* (Willd.) Ohwi root is a traditional medicinal resource rich in bioactive isoflavonoids with known antioxidant and anti-inflammatory properties. However, the chemical composition and biological activities of *P. lobata* root extracts can vary depending on the extraction solvent. In this study, we systematically compared *P. lobata* root extracts prepared using water, ethanol (30%, 70%, and 100%), and methanol to evaluate the effects of solvent selection on extraction yield, HPLC-based chemical profiles of major isoflavonoids, antioxidant capacity, and cellular responses in vitro. Chemical characterization by HPLC revealed distinct solvent-dependent differences in the relative abundance of key isoflavonoids, including puerarin, daidzin, and daidzein, defining characteristic chemical profiles for each extract. Antioxidant activity was evaluated using DPPH and ABTS radical scavenging assays, along with measurements of total polyphenol and flavonoid content. Cell viability was examined in HeLa cells using an MTT assay to define non-cytotoxic concentration ranges. The anti-inflammatory potential of the extracts was further assessed by measuring TNF-α-induced secretion of pro-inflammatory cytokines in HeLa cells. The results revealed marked solvent-dependent differences in extraction yield, chemical composition, and functional activity. Notably, methanol and ethanol extracts exhibited enriched isoflavonoid profiles associated with enhanced antioxidant and anti-inflammatory responses. Overall, this integrated chemical and functional evaluation demonstrates that solvent selection plays a critical role in determining the chemical characteristics and bioactivity of *P. lobata* root extracts. These findings provide a basis for rational solvent selection in the preparation of plant-derived extracts and support the potential use of *P. lobata* root as a functional source of antioxidant and anti-inflammatory compounds.

## 1. Introduction

*Pueraria lobata* (Willd.) Ohwi is a traditional medicinal plant widely used in East Asia. The roots of *P. lobata* have long been used for the treatment of numerous diseases, including fever, diarrhea, gastrointestinal disorders, and inflammatory conditions [1]. Phytochemical studies have demonstrated that *P. lobata* roots are rich in isoflavonoids, such as puerarin, daidzin, and daidzein, which are associated with various biological activities, including antioxidant, anti-inflammatory, and metabolic regulatory effects [2].

The biological activities of plant-derived extracts are influenced by extraction conditions, particularly the solvent used. Different solvents vary in polarity and extraction efficiency, resulting in marked differences in yield, chemical composition, and functional properties. Commonly used solvents for extraction include water, ethanol, and methanol [3]. Water is a polar solvent and the safest extraction method, often employed for extracting water-soluble components [4]. Ethanol is also a polar solvent that can extract a variety of compounds, making it effective for extracting bioactive compounds and suitable for use in food and pharmaceutical applications [5]. Ethanolic extracts of *P. lobata* have been reported to efficiently recover isoflavone glycosides such as puerarin and daidzin, as well as other phenolic constituents. Methanol is an effective polar solvent for extracting various polar compounds, but its toxicity necessitates caution [6]. Methanolic extraction is often associated with higher recovery of total phenolics and flavonoids, including daidzein and related aglycones. Given that solvent polarity critically determines the qualitative and quantitative composition of extracted phytochemicals, comparative evaluation of solvents with distinct polarities is essential for understanding differences in biological activity. While numerous previous studies have focused on the pharmacological effects of individual compounds isolated from *P. lobata*, comparative analyses of *P. lobata* root extracts prepared using different solvents are lacking. Previous studies have reported antioxidant, anti-inflammatory, and metabolic effects of individual compounds or specific solvent extracts of *P. lobata*. However, most investigations have focused on isolated constituents or single extraction conditions rather than performing a systematic comparison of extracts prepared using solvents with distinct polarities. Furthermore, integrated evaluation of extraction yield, phytochemical composition, radical scavenging activity, cytotoxicity, and TNF-α-induced inflammatory responses within the same experimental framework remains limited. Therefore, the present study provides a comprehensive and comparative assessment of solvent-dependent differences in both chemical profiles and biological activities of *P. lobata* root extracts. Therefore, we aimed to analyze the optimal solvents and functional efficacy for extracting important components from *P. lobata* roots.

Oxidative stress and inflammation are pathological responses closely linked to various chronic diseases [7]. Antioxidant substances can mitigate oxidative damage, while anti-inflammatory substances suppress the excessive production of pro-inflammatory mediators. In this context, plant extracts rich in polyphenols and flavonoids, which exhibit antioxidant and anti-inflammatory properties, have attracted considerable attention as multifunctional natural resources capable of modulating both oxidative and inflammatory responses [8,9]. Therefore, a systematic evaluation of antioxidant and anti-inflammatory activities is essential to understanding the functional potential of plant extracts and establishing a foundation for application.

In vitro screening is used to assess the biological activity of compounds in plant extracts and analyze extraction conditions. Chemical profiling methods, such as high-performance liquid chromatography (HPLC), identify key bioactive components, while antioxidant assays, including 2,2-diphenyl-1-picrylhydrazyl (DPPH) and ABTS radical scavenging assays, provide information on radical scavenging capacity [10]. Furthermore, cell-based assays can evaluate cytotoxicity and inflammatory responses under controlled conditions. The human cervical cancer HeLa cells we used are widely recognized as an epithelial cell model for studying pathways activated by pro-inflammatory stimuli such as tumor necrosis factor-α (TNF-α) [11].

The present study aimed to systematically compare *P. lobata* root extracts prepared using different solvents, including water, ethanol, and methanol. We evaluated extraction yield, chemical composition, and antioxidant capacity, and investigated the effects of the extracts on cell viability and TNF-α-induced inflammatory cytokine production in HeLa cells. Therefore, this study seeks to provide a comprehensive framework for understanding the differences between solvents and to establish a scientific basis for the development and utilization of *P. lobata* root extract as a functional plant-derived resource.

## 2. Results

### 2.1. Extraction Yield and HPLC Analysis of P. lobata Root Extracts

**Figure 1:**
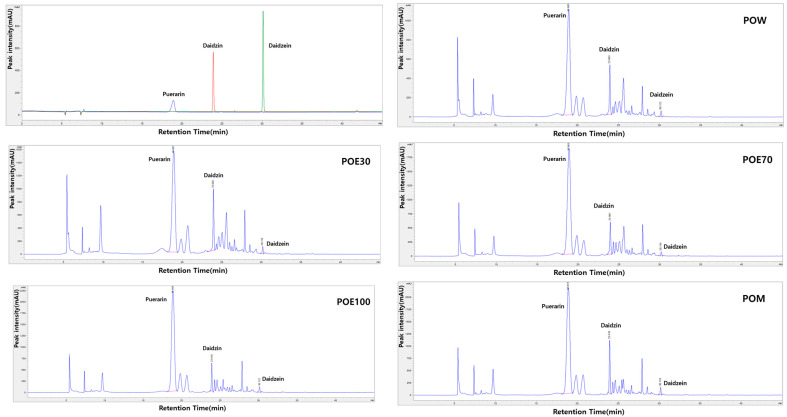
Comparative HPLC analysis of *P. lobata* root extracts prepared using different solvents. Representative HPLC chromatograms of standard compounds (puerarin, daidzin, and daidzein) and *P. lobata* root extracts obtained using various extraction solvents are shown. Chromatograms of the POW, POE30, POE70, POE100, and POM illustrate distinct solvent-dependent chemical profiles, reflected by differences in the relative abundance of major isoflavonoids. POW: water extract, POE30: 30% ethanol extract, POE70: 70% ethanol extract, POE100: 100% ethanol extract, POM: methanol extract.

To investigate solvent-dependent differences in the chemical profiles of *P. lobata* root extracts, HPLC analysis was performed on extracts prepared using water (POW), 30% ethanol (POE30), 70% ethanol (POE70), 100% ethanol (POE100), and methanol (POM). The extraction yield varied markedly depending on the solvent, with POW showing the highest yield (31.67%), followed by POE30 (22.27%), POE70 (21.16%), and POE100 (21.90%), whereas POM exhibited a substantially lower yield (2.17%), indicating clear solvent-dependent extraction efficiency. Representative HPLC chromatograms of the different solvent extracts are shown in Figure 1. Comparative analysis revealed distinct solvent-dependent chemical profiles, characterized by differences in the relative abundance of major isoflavonoids. HPLC analysis revealed distinct peaks corresponding to puerarin, daidzin, and daidzein at retention times of 18.61 min, 23.87 min, and 30.15 min, respectively. Puerarin, daidzin, and daidzein were identified by comparison with authentic standards and were detected in all extracts; however, their relative peak intensities and concentrations varied markedly according to the extraction solvent. Notably, the methanol extract (POM) exhibited the highest overall levels of both glycosylated and aglycone isoflavones, including puerarin, daidzin, and daidzein, indicating its strong extraction efficiency for isoflavone compounds. Among the ethanol-based extracts, increasing ethanol concentration was associated with a gradual increase in puerarin content, with POE100 showing a higher abundance compared to POE30 and POE70. In contrast, the aqueous extract (POW) contained comparatively lower levels of these isoflavonoids, suggesting preferential extraction of more polar, non-isoflavone constituents. Quantitative determination of puerarin, daidzin, and daidzein confirmed these solvent-dependent differences and is summarized in Table 1. Collectively, these results demonstrate that extraction solvent selection critically influences both the extraction yield and the chemical characteristics of *P. lobata* root extracts.

### 2.2. DPPH Radical Scavenging Activity of P. lobata Root Extracts

**Figure 2:**
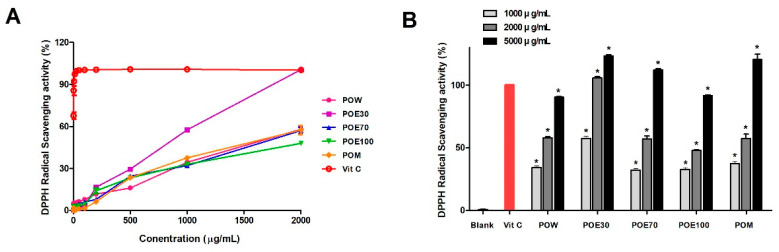
DPPH radical scavenging profiles of *P. lobata* root extracts obtained using different solvents. (**A**) Concentration-dependent DPPH radical scavenging activities of *P. lobata* root extracts prepared using various extraction solvents. The POW, POE30, POE70, POE100, and POM were evaluated across a range of concentrations, with Vitamin C used as a positive control. (**B**) Comparison of DPPH radical scavenging activities of the extracts at various concentrations (1000, 2000, and 5000 μg/mL). Data are presented as the mean ± standard error of the mean (SEM) from a minimum of three independent experiments. * *p* < 0.05 compared with the blank control. POW: water extract, POE30: 30% ethanol extract, POE70: 70% ethanol extract, POE100: 100% ethanol extract, POM: methanol extract.

DPPH is widely used as a stable free radical to evaluate the antioxidant capacity of natural compounds and plant extracts [12]. The DPPH radical scavenging activities of different solvent extracts of *P. lobata* roots at various concentrations are shown in Figure 2. All extracts exhibited a concentration-dependent increase in DPPH radical scavenging activity (Figure 2A). Among the different solvent extracts, POE30 demonstrated the highest scavenging activity across the tested concentration range. At higher concentrations (5 mg/mL), the DPPH radical scavenging activities of all extracts were comparable to that of the positive control, vitamin C; however, their scavenging efficiencies remained lower than that of vitamin C at the same concentration (Figure 2B). These results indicate that the antioxidant capacity of *P. lobata* root extracts is strongly influenced by the extraction solvent, with POE30 showing the most potent DPPH radical scavenging activity. To further compare antioxidant potency among extracts, IC_50_ values were estimated from the dose–response curves. POE30 exhibited the lowest IC_50_ (~840 μg/mL), indicating the strongest DPPH radical scavenging activity, whereas POW, POE70, POE100, and POM showed markedly higher IC_50_ values (>1500 μg/mL). These findings demonstrate solvent-dependent differences in DPPH scavenging activity.

### 2.3. ABTS Radical Scavenging Activity of P. lobata Root Extracts

**Figure 3:**
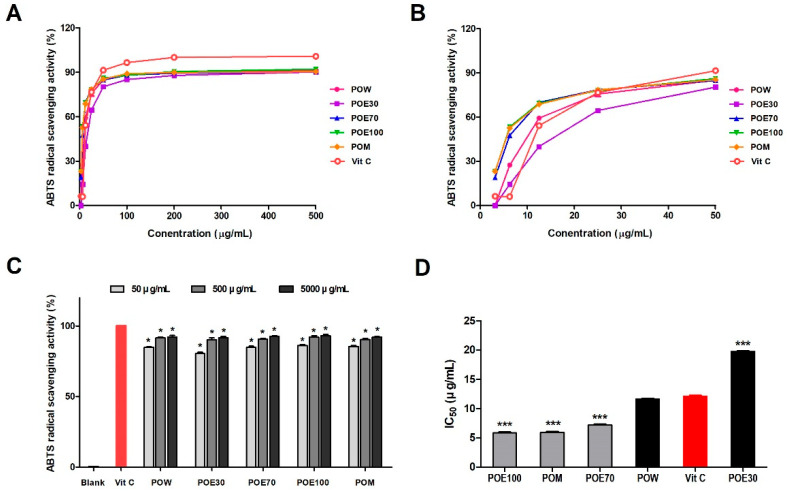
ABTS radical scavenging profiles of *P. lobata* root extracts obtained using different solvents. (**A**) Concentration-dependent ABTS radical scavenging activities of *P. lobata* root extracts prepared using various extraction solvents. The POW, POE30, POE70, POE100, and POM were evaluated across a range of concentrations, with Vitamin C used as a positive control. (**B**) Expanded view of the low concentration range (3.13–50 μg/mL), highlighting differences in antioxidant potency among the extracts prior to saturation. (**C**) Comparison of ABTS radical scavenging activities of the extracts at selected concentrations (50, 500, and 5000 μg/mL). (**D**) IC_50_ values calculated from the concentration–response curves, indicating the concentration required to achieve 50% ABTS radical scavenging. Lower IC_50_ values indicate higher antioxidant potency. Data are presented as the mean ± SEM from a minimum of three independent experiments. * *p* < 0.05 compared with the blank control. *** *p* < 0.001 compared with the Vit C. POW: water extract, POE30: 30% ethanol extract, POE70: 70% ethanol extract, POE100: 100% ethanol extract, POM: methanol extract.

ABTS is a widely used assay for evaluating antioxidant capacity, in which ABTS radical cations generated by reaction with potassium persulfate are scavenged by antioxidant compounds [13]. The ABTS radical scavenging activities of different solvent *P. lobata* root extracts at varying concentrations are shown in Figure 3. All extracts exhibited a concentration-dependent increase in ABTS radical scavenging activity (Figure 3A). Regardless of the extraction solvent, the scavenging activity increased rapidly at lower concentrations and gradually plateaued at higher concentrations. To enable a more accurate comparison of antioxidant potency prior to saturation, the low concentration range (3.13–50 μg/mL) was further examined (Figure 3B). In this range, the extracts exhibited distinct differences in scavenging activity depending on the extraction solvent, with ethanol and methanol extracts generally showing higher activity than the water extract. At the highest tested concentration, all *P. lobata* root extracts demonstrated ABTS radical scavenging activities comparable to those of the positive control, vitamin C (Figure 3C). These results indicate that the ABTS radical scavenging capacity of *P. lobata* root extracts is consistent across different solvents and becomes more active at higher concentrations. Furthermore, IC_50_ values calculated from the concentration–response curves confirmed these differences in antioxidant potency, with POE100 and POM exhibiting lower IC_50_ values compared to POW and POE30, indicating stronger radical scavenging activity (Figure 3D).

### 2.4. Total Polyphenol and Flavonoid Contents of P. lobata Root Extracts

**Figure 4:**
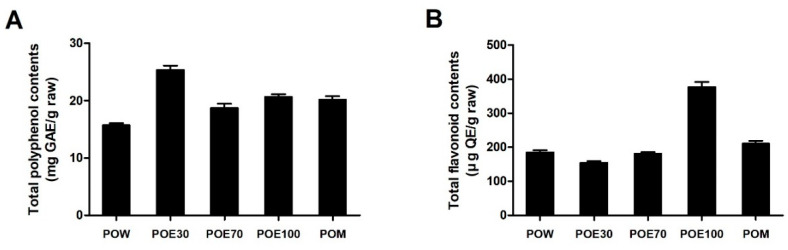
Comparative analysis of total polyphenol and flavonoid contents in *P. lobata* root extracts obtained using different solvents. (**A**) TPC of *P. lobata* root extracts prepared using various extraction solvents, expressed as mg gallic acid equivalents (GAE) per g of raw material. (**B**) TFC of *P. lobata* root extracts, expressed as μg quercetin equivalents (QE) per g of raw material. Data are presented as the mean ± SEM from a minimum of three independent experiments. POW: water extract, POE30: 30% ethanol extract, POE70: 70% ethanol extract, POE100: 100% ethanol extract, POM: methanol extract.

The total polyphenol content (TPC) and total flavonoid content (TFC) of *P. lobata* root extracts prepared using different solvents are shown in Figure 4. When normalized to the raw material weight, TPC varied depending on the extraction solvent (Figure 4A). Among the *P. lobata* root extracts, the POE30 exhibited the highest TPC, followed by the POE100 and POM extracts, whereas the POW showed the lowest polyphenol content. In contrast to the TPC trend, TFC displayed a different solvent-dependent profile (Figure 4B). The POE100 had the highest flavonoid content among the extracts, while the other extracts showed comparatively lower TFC values. Additionally, Table 2 summarizes the extraction yields together with TPC and TFC values expressed on both an extract-weight basis and a raw-material basis to account for differences in extraction efficiency among solvents and to avoid misinterpretation caused by concentration effects. Collectively, these results demonstrate that the extraction solvent differentially affects the content of polyphenols and flavonoids from *P. lobata* roots.

### 2.5. Cell Viability of P. lobata Root Extracts in HeLa Cells

**Figure 5:**
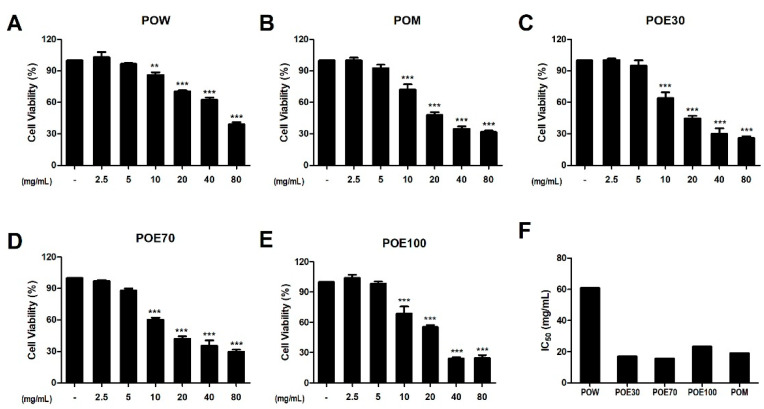
Effects of *P. lobata* root extracts on HeLa cell viability. HeLa cells were treated with increasing concentrations of *P. lobata* root extracts prepared using different extraction solvents, and cell viability was assessed using the MTT assay. (**A**) POW, (**B**) POM, (**C**) POE30, (**D**) POE70, and (**E**) POE100. Cell viability is expressed as a percentage relative to the untreated control. (**F**) IC_50_ values were estimated from interpolated dose–response curves. Data are presented as the mean ± SEM from a minimum of three independent experiments. ** *p* < 0.01 and *** *p* < 0.001 compared with the blank control. POW: water extract, POE30: 30% ethanol extract, POE70: 70% ethanol extract, POE100: 100% ethanol extract, POM: methanol extract.

The effects of *P. lobata* root extracts prepared with different extraction solvents on HeLa cell viability were evaluated using the 3-(4,5-dimethylthiazol-2-yl)-2,5-diphenyltetrazolium bromide (MTT) assay. As shown in Figure 5A–E, treatment with all extracts led to a concentration-dependent reduction in cell viability. Notably, significant decreases were observed for all extracts at concentrations of 10 mg/mL and higher, indicating a clear dose-dependent cytotoxic effect. Furthermore, statistical comparison between extracts at 10 mg/mL, where cytotoxic effects became evident, revealed that differences in cytotoxic potency were limited, with significant differences observed only in selected comparisons (e.g., POW vs. POE70), while most extracts exhibited broadly comparable inhibitory effects on HeLa cell viability. Collectively, these results demonstrate that *P. lobata* root extracts exert solvent-dependent inhibitory effects on HeLa cell growth, supporting their potential utility for preliminary cytotoxicity screening.

### 2.6. Regulation of TNF-α-Induced Inflammatory Cytokine Expression by P. lobata Root Extracts in HeLa Cells

**Figure 6:**
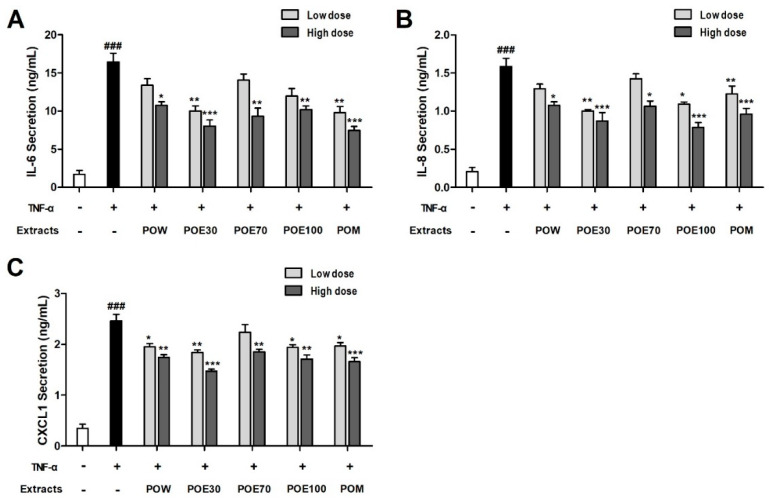
Effects of *P. lobata* root extracts on TNF-α-induced inflammatory cytokine secretion in HeLa cells. HeLa cells were pretreated with low or high doses of *P. lobata* root extracts, followed by stimulation with TNF-α. The levels of secreted inflammatory cytokines (**A**) IL-6, (**B**) IL-8, and (**C**) CXCL1 were measured by enzyme-linked immunosorbent assay (ELISA). Data are presented as the mean ± SEM from a minimum of three independent experiments. ^###^ *p* < 0.001 compared with the control. * *p* < 0.05, ** *p* < 0.01, and *** *p* < 0.001 compared with the TNF-α treatment. POW: water extract, POE30: 30% ethanol extract, POE70: 70% ethanol extract, POE100: 100% ethanol extract, POM: methanol extract.

To evaluate the anti-inflammatory potential of *P. lobata* root extracts, HeLa cells were pretreated with low (2.5 mg/mL) and high (5 mg/mL) doses of various *P. lobata* root extracts, followed by stimulation with TNF-α. The secretion levels of pro-inflammatory cytokines were subsequently measured. Cytokine measurements were performed using concentrations previously confirmed to be non-cytotoxic based on the cell viability assay. As shown in Figure 6A–C, TNF-α stimulation markedly increased the secretion of IL-6, IL-8, and CXCL1 compared to the control. Pretreatment with all *P. lobata* root extracts significantly attenuated TNF-α-induced cytokine production in a dose-dependent manner. At the higher concentration (5 mg/mL), all extracts showed substantial suppression of IL-6, IL-8, and CXCL1 production relative to TNF-α-treated cells. Statistical analysis indicated that most extracts produced broadly comparable reductions in cytokine secretion under the tested conditions. Statistical comparisons between extracts revealed that differences were limited and observed only in selected comparisons, with most extracts demonstrating broadly comparable anti-inflammatory effects at the tested concentrations. Collectively, these results indicate that *P. lobata* root extracts effectively suppress TNF-α-induced inflammatory responses in HeLa cells, and that this anti-inflammatory effect depends on both the extraction solvent and treatment concentration.

## 3. Discussion

In this study, we compared *P. lobata* root extracts prepared using different solvents and evaluated their chemical profiles, antioxidant capacity, and biological activities in vitro. By integrating extraction yield, phytochemical composition, antioxidant effects, and cellular responses, our findings offer a comprehensive overview of how solvent selection influences both the chemical profile and functional potential of *P. lobata* root extracts.

The extraction yield varied significantly depending on the solvents, with the water extract showing the highest yield and the methanol extract exhibiting a considerably lower yield. These differences align with previous studies indicating that highly polar solvents efficiently extract hydrophilic components, whereas organic solvents may selectively enrich specific secondary metabolites despite lower overall recovery [14,15]. In addition, although extraction yields varied considerably depending on solvent polarity, the phenolic and flavonoid contents represent only a fraction of the total extractable solids. *P. lobata* roots contain substantial amounts of carbohydrates and other non-phenolic constituents, which likely contributed to differences in overall extraction yield without proportionally affecting phenolic concentration. Importantly, although the methanol extract displayed high polyphenol and flavonoid contents when expressed on an extract-weight basis, this effect was largely attributable to its low extraction yield, resulting in a concentration effect. Therefore, it is essential to evaluate phytochemical contents using both raw material-based and extract-based normalization to accurately determine functional relevance.

*P. lobata* root is one of the oldest medicinal plants used in ancient China [16]. *P. lobata* root extract contains various bioactive compounds, including puerarin, daidzin, and daidzein [17]. These compounds have been reported to exhibit antioxidant, anti-inflammatory, and antimicrobial activities [18]. Puerarin, daidzin, and daidzein (major isoflavonoid compounds of *P. lobata*) were detected in all extracts but at different relative levels. HPLC analysis demonstrated that the solvent influenced the chemical composition of the extracts. Notably, ethanol extracts exhibited a higher content of these bioactive constituents compared with the water extract, supporting the suitability of ethanol solvents for extracting isoflavonoids from *P. lobata* root.

DPPH and ABTS free radical scavenging assays are widely used and established methods for evaluating the antioxidant capacity of plant extracts, as they can rapidly measure free radical scavenging activity through different reaction mechanisms [13,19]. The antioxidant activities of the *P. lobata* root extracts, assessed using DPPH and ABTS radical scavenging assays, showed solvent-dependent differences. While all extracts demonstrated concentration-dependent radical scavenging activity, the POE30 exhibited superior activity, particularly in the DPPH assay. Additionally, ABTS scavenging activity approached that of the positive control at higher concentrations for all extracts. These results highlight the importance of utilizing multiple antioxidant assays to better differentiate the capacities of various extracts. The lack of a strict correlation between total phenolic/flavonoid content and radical scavenging activity suggests that antioxidant potency depends more on the qualitative composition and structural characteristics of individual constituents than on total quantity alone. Comparable observations have been reported in previous studies on plant extracts, where antioxidant activity was influenced more strongly by the specific composition and structural features of phenolic compounds than by their total concentration [8]. Minor components and potential synergistic interactions may also contribute to the observed activity, particularly in the POE30.

TPC and TFC are commonly determined to estimate the overall abundance of phenolic and flavonoid compounds in plant extracts, recognized as major contributors to antioxidant activity [20,21]. We examined the antioxidant properties of *P. lobata* root extracts by measuring TPC and TFC. The relatively high polyphenol content of POE30 and the high flavonoid content of POE100 suggest that different solvents selectively extract specific compounds. However, the absence of a direct correlation between TPC, TFC, and free radical scavenging activity indicates that antioxidant capacity is influenced not only by the total content of plant compounds but also by their composition. Antioxidant capacity is determined not only by the total amount of phenolic or flavonoid compounds but also by their qualitative composition. Radical scavenging efficiency depends on structural features such as hydroxyl group positioning, conjugation, and glycosylation, which influence electron-donating ability and redox potential. For example, isoflavone glycosides may exhibit different radical scavenging kinetics compared to their aglycone counterparts. Furthermore, interactions among co-extracted compounds may result in synergistic or antagonistic effects, thereby modulating the overall antioxidant response. Therefore, extracts with similar total phenolic content may display comparable radical scavenging activity despite differences in individual constituent profiles.

To further examine these findings in terms of their biological relevance, we evaluated the effects of *P. lobata* root extracts on HeLa cell viability as part of a cytotoxicity screening. Consequently, all *P. lobata* root extracts reduced cell viability in a concentration-dependent manner. Importantly, these effects were used to define non-cytotoxic concentration ranges for subsequent functional assays rather than to confirm anticancer activity.

TNF-α is a key pro-inflammatory cytokine that activates epithelial inflammatory signaling primarily through the NF-κB pathway, leading to the transcriptional upregulation of downstream cytokines and chemokines such as IL-6, IL-8, and CXCL1 [22]. This TNF-α-driven inflammatory response in epithelial cells is widely used as an in vitro model to evaluate the anti-inflammatory potential of bioactive compounds. Based on the established non-cytotoxic low and high concentrations, we further investigated the anti-inflammatory potential of *P. lobata* root extracts using TNF-α–stimulated HeLa cells. TNF-α markedly induced the secretion of pro-inflammatory cytokines, including IL-6, IL-8, and CXCL1, whereas pretreatment with *P. lobata* root extracts significantly attenuated this response in a dose-dependent manner. These findings suggest that *P. lobata* root extracts can modulate TNF-α–stimulated inflammatory signaling pathways in epithelial cells through the suppression of downstream cytokine production. Consistent with our observations, several studies have reported that isoflavonoids such as puerarin can suppress inflammatory signaling pathways including NF-κB and MAPK, leading to reduced production of pro-inflammatory cytokines [18,23]. The observed differences in anti-inflammatory activity among extracts may be attributed to qualitative differences in phytochemical composition rather than total phenolic or flavonoid content alone. Individual isoflavones and phenolic constituents can differentially regulate key inflammatory signalling pathways, including NF-κB and MAPK cascades, which are central to TNF-α-induced cytokine expression. Variations in compound polarity and extraction solvent may alter the relative abundance of bioactive molecules capable of interfering with these pathways. Furthermore, interactions among co-extracted constituents may result in synergistic or modulatory effects that influence the overall suppression of IL-6, IL-8, and CXCL1 production. Therefore, differences in solvent extraction likely led to distinct phytochemical profiles that contributed to the differential biological responses observed in HeLa cells.

HPLC analysis in the present study focused on the identification and quantification of representative isoflavonoid markers (puerarin, daidzin, and daidzein) to enable comparative evaluation of solvent-dependent chemical profiles. In addition to these identified compounds, additional chromatographic peaks were observed, which may correspond to other isoflavones and phenolic constituents previously reported in *P. lobata*, such as genistein, and formononetin. Although these minor peaks were not individually characterized in this study, their presence is consistent with the known phytochemical diversity of *P. lobata* extracts. The current work was designed to perform comparative profiling rather than comprehensive structural elucidation, and therefore focused on well-established marker compounds to ensure reliable quantification and reproducibility.

Taken together, our results indicate that solvent selection plays a critical role in determining the chemical composition and biological activity of *P. lobata* root extracts. The observed activities varied depending on the extraction solvent and the specific biological assay. These findings highlight that the functional properties of *P. lobata* extracts are strongly influenced by solvent-dependent phytochemical composition.

## 4. Materials and Methods

### 4.1. Chemical Reagents

Puerarin, daidzin, and daidzein, vitamin C, gallic acid, quercetin, dimethyl sulfoxide (DMSO), and MTT solution were obtained from Sigma–Aldrich (St. Louis, MO, USA). TNF-α was purchased from BioLegend (San Diego, CA, USA). Dulbecco’s modified Eagle’s medium (DMEM) and fetal bovine serum (FBS) were obtained from Gibco BRL (Invitrogen Co., Waltham, MA, USA).

### 4.2. Plant Material and Preparation of P. lobata Root Extract

The roots of *P. lobata* used in this study were obtained from the Gwangmyeongdang Oriental Medicine Pharmaceutical Company (Ulsan, Republic of Korea). The plant material had been previously authenticated by Professor Yong-Deok Jeon at Woosuk University, and a voucher specimen (No. 2024-WSKP07) has been deposited at the Department of Oriental Pharmacy, Woosuk University. The extraction procedure was conducted according to our previously reported method [23]. Briefly, dried roots of *P. lobata* were pulverized into a fine powder, and 50 g of the powder was extracted with 500 mL of various solvents, including methanol, 30% ethanol, 70% ethanol, 100% ethanol, and distilled water. All solvents were refluxed at 75 °C for 3 h using a heating mantle. After extraction, the mixtures were filtered through Advantec filter paper, and the filtrates were concentrated under reduced pressure using a rotary evaporator. The concentrated extracts were frozen at −75 °C for 24 h and subsequently freeze-dried at −80 °C under vacuum (100 mTorr) for 72 h to obtain dry extracts. All dried extracts were stored at 4 °C until further analysis. After freeze-drying, the extracts were dried to constant weight and the extraction yield (%) was calculated as the weight of the dried extract relative to the initial dry root powder used for extraction.

### 4.3. HPLC Analysis of P. lobata Root Extracts

The chromatographic conditions for HPLC analysis are summarized in Table 3. Additional UHPLC–PDA chromatograms and spectral data are provided in the Supplementary Materials.

### 4.4. Antioxidant Activity

#### 4.4.1. DPPH Radical Scavenging Assay

The antioxidant activity of various *P. lobata* root extracts was evaluated by measuring their ability to scavenge DPPH radicals. The method followed a previously established protocol [23]. Briefly, different *P. lobata* root extracts or vitamin C (used as a positive control) were added to 2 mL of a 0.15 mM DPPH solution. The mixtures were vortexed for 10 s to ensure thorough mixing and then incubated for 30 min at room temperature in the dark. After incubation, the absorbance of the reaction mixtures was measured at 517 nm using a microplate reader (BioTek Instruments; Winooski, VT, USA). The DPPH radical scavenging activity was calculated using the following equation:

DPPH radical scavenging activity (%) = {1 − (Abs_sample/Abs_control)} × 100

‘Abs_control’ was defined as the absorbance of the DPPH working solution mixed with the same volume of extraction solvent (without sample), incubated under identical conditions. Vitamin C was a positive control and measured independently.

#### 4.4.2. ABTS Radical Cation Decolorization Assay

The ABTS radical cation scavenging activity of various *P. lobata* root extracts was determined using the ABTS assay. The method followed a previously established protocol [24]. Briefly, a 7.4 mM ABTS solution was mixed with 2.6 mM potassium persulfate at a 1:1 (*v*/*v*) ratio and allowed to react for 24 h at room temperature to generate the ABTS solution. The resulting solution was adjusted to pH 7.4 prior to use. In a 96-well microplate, 20 μL of each *P. lobata* root extract was mixed with 180 μL of the ABTS radical solution and incubated at room temperature for 10 min. The absorbance was then measured at 734 nm using a microplate reader (BioTek Instruments; Winooski, VT, USA). The ABTS radical scavenging activity was calculated using the following equation:

ABTS radical scavenging activity (%) = {1 − (Abs_sample/Abs_control)} × 100

‘Abs_control’ was defined as the absorbance of the ABTS working solution mixed with the same volume of extraction solvent (without sample), incubated under identical conditions. Vitamin C was a positive control and measured independently.

### 4.5. TPC Determination

The TPC of the *P. lobata* root extracts was determined using the Folin–Ciocalteu colorimetric method. Gallic acid was used as a standard for calibration. A gallic acid stock solution (1 mg/mL) was prepared in 70% methanol and diluted to obtain standard solutions in the concentration range of 10–500 μg/mL. For the assay, 100 μL of each extract sample or standard solution was mixed with 50 μL of Folin–Ciocalteu’s phenol reagent (2 N) and 500 μL of deionized water. The mixture was vortexed and incubated at room temperature for 5 min. Subsequently, 600 μL of 2% sodium bicarbonate (NaHCO_3_, *w*/*v*) solution was added, and the reaction mixture was incubated at room temperature or 37 °C for 30–60 min. After incubation, the reaction mixtures were centrifuged to remove any precipitates, and aliquots (≥200 μL) of the supernatants were transferred to a 96-well microplate. The absorbance was measured at 765 nm using a microplate reader (BioTek Instruments; Winooski, VT, USA). The TPC was calculated from the calibration curve and expressed as micrograms of gallic acid equivalents per gram of extract (μg GAE/g). The TPC (μg/g) was calculated using the following equation:

TPC (μg GAE/g) = (*C* × *V* × *D*)/*W*

*C* is the concentration determined from the calibration curve (μg/mL), *V* is the volume of the extraction solvent (mL), *D* is the dilution factor, and *W* is the weight of the raw material (g).

### 4.6. TFC Determination

The TFC of the *P. lobata* root extracts was determined using an aluminum chloride (AlCl_3_) colorimetric method. Quercetin was used as the reference compound for calibration. A stock solution of the standard (1 mg/mL) was prepared in 70% methanol and serially diluted to generate standard solutions in the range of 1–50 μg/mL. A 10% aluminum chloride solution was prepared by dissolving aluminum chloride hexahydrate (AlCl_3_•6H_2_O) in distilled water (10% *w*/*v*) and stored at 4 °C. For the assay, each extract sample solution or standard solution was mixed with the 10% AlCl_3_ solution at a 1:1 (*v*/*v*) ratio and vortexed thoroughly. The mixtures were incubated at room temperature for 5 min and then centrifuged to remove any particulates. Aliquots (≥200 μL) of the supernatants were transferred to a 96-well microplate, and absorbance was measured at 415 nm using a microplate reader (BioTek Instruments; Winooski, VT, USA). The TFC was calculated from the calibration curve and expressed as micrograms of quercetin equivalents per gram of sample (μg QE/g). TFC was calculated using the following equation:

TFC (μg QE/g) = (*C* × *V* × *D*)/*W*

C is the concentration obtained from the calibration curve (μg/mL), V is the volume of the extraction solvent (mL), D is the dilution factor, and W is the weight of the raw material (g).

### 4.7. Cell Culture

Human cervical carcinoma HeLa cells were obtained from the Korean Cell Line Bank (Seoul, Republic of Korea; KCLB No:10002). The cells were cultured in DMEM supplemented with 10% (*v*/*v*) heat-inactivated FBS, 100 U/mL penicillin, and 100 μg/mL streptomycin. The cell cultures were incubated at 37 °C in a humidified atmosphere containing 5% CO_2_ and were routinely subcultured to maintain exponential growth.

### 4.8. Cell Viability Assay (MTT)

Cell viability was assessed using the MTT assay. HeLa cells were seeded into 96-well plates at 1 × 10^4^ cells/well and allowed to attach overnight. The cells were then treated with different *P. lobata* root extracts at the indicated concentrations for 24 h. Following treatment, 20 µL of MTT solution (0.5 mg/mL) was added to each well, and the cells were incubated at 37 °C to allow for the formation of formazan crystals. After incubation, the culture medium was carefully removed, and the formazan crystals were dissolved in 150 µL of DMSO. The absorbance was measured at 570 nm using a microplate reader (BioTek Instruments; Winooski, VT, USA). Cell viability was determined in triplicate and expressed as a percentage of the untreated control.

### 4.9. ELISA

HeLa cells were seeded in culture plates and allowed to adhere overnight. The cells were pretreated for 1 h with *P. lobata* root extracts (both low and high doses) and subsequently stimulated with TNF-α (10 ng/mL) for 24 h. After incubation, the culture supernatants were collected and clarified by centrifugation to remove cell debris. The secretion levels of IL-6, IL-8, and CXCL1 in the supernatants were quantified using ELISA kits according to the manufacturers’ instructions. Absorbance was measured using a microplate reader (BioTek Instruments; Winooski, VT, USA) at 450 nm.

### 4.10. Statistical Analysis

All statistical analyses were conducted using GraphPad Prism (version 5.0; GraphPad Software, San Diego, CA, USA). Data are expressed as the mean ± standard error of the mean (SEM) and were obtained from at least three independent experiments. Comparisons among multiple groups were performed using one-way analysis of variance (ANOVA), followed by Tukey’s post hoc test. Differences were considered statistically significant at *p* < 0.05.

## 5. Conclusions

In conclusion, the extraction solvent critically influenced the chemical composition and biological activities of *P. lobata* root extracts. HPLC analysis confirmed solvent-dependent variations in major isoflavonoids, including puerarin, daidzin, and daidzein. All extracts exhibited concentration-dependent antioxidant activity, while ethanol extracts (POE30 and POE70) showed comparatively stronger cytotoxic effects in HeLa cells, with estimated IC_50_ values of approximately 17–16 mg/mL. Moreover, treatment at 2.5–5 mg/mL significantly attenuated TNF-α-induced inflammatory cytokine production. Taken together, these findings provide a comprehensive framework linking solvent-dependent chemical profiles to functional biological outcomes and offer a scientific basis for optimizing extraction conditions for *P. lobata* root preparations.

## Figures and Tables

**Table 1:** Contents of major isoflavonoids in *P. lobata* root extracts, used to characterize solvent-dependent differences in chemical composition. Values are expressed as mean ± SD (n = 3).

**Contents of Major Isoflavonoids (mg/g)**			
**Extract**	**Puerarin**	**Daidzin**	**Daidzein**
POW	267.4 ± 30.10	45.88 ± 5.88	2.47 ± 0.34
POE30	361.0 ± 30.55	76.35 ± 6.34	5.73 ± 0.33
POE70	430.7 ± 25.36	51.64 ± 3.95	2.56 ± 0.33
POE100	485.6 ± 13.83	50.59 ± 1.39	4.52 ± 0.51
POM	508.4 ± 15.02	92.04 ± 8.50	6.79 ± 0.25

**Table 2:** Extraction yield and extract-based polyphenol and flavonoid contents of *P. lobata* root extracts. Values are expressed as mean ± SD (n = 3). TPC, total phenolic content; TFC, total flavonoid content.

Extract	Yield (%)	TPC (mg GAE/ g Extract)	TPC (mg GAE/ g Dry Root)	TFC (mg QE/ g Extract)	TFC (mg QE/ g Dry Root)
POW	31.67	49.6 ± 2.1	15.7 ± 0.7	582.2 ± 33.0	184.4 ± 10.5
POE30	22.27	113.8 ± 6.1	25.3 ± 1.4	691.9 ± 39.1	154.1 ± 8.7
POE70	21.16	88.5 ± 6.0	18.7 ± 1.3	858.8 ± 14.8	181.7 ± 3.1
POE100	21.90	94.5 ± 3.5	20.7 ± 0.8	1628.2 ± 58.0	356.6 ± 12.7
POM	2.17	930.0 ± 50.3	20.2 ± 1.1	9691.2 ± 691.2	210.3 ± 15.0

**Table 3:** HPLC analytical conditions.

HPLC Instrument	Agilent 1200 series		
Detector	DAD 260 nm		
Column	Aegispak-L C18 (4.6 × 150 mm I.D., 3.0 μm)		
Column temperature	35 °C		
Injection volume	15 μL		
Flow rate	0.32 mL/min		
Mobile phase	Solvent A: 0.1% aqueous formic acid, Solvent B: acetonitrile		
	Time	A	B
	Initiation	85	15
	11	85	15
	13	80	20
	17	70	30
	23	55	45
	31	40	60
	38	20	80
	40	20	80
	50	85	15

## Data Availability

The data are included within the article and Supplementary Materials.

## Supplementary Materials

The following supporting information can be downloaded at https://www.mdpi.com/article/10.3390/molecules31060965/s1, Figure S1: Three-dimensional UHPLC–PDA spectral maps (200–400 nm) of different solvent extracts, illustrating the overall UV fingerprint profiles, Figure S2: UHPLC–PDA chromatogram (254 nm) of the reference standard puerarin, Figure S3: UHPLC–PDA chromatogram (254 nm) of the reference standard daidzin, Table S1. Compound component analysis of *P. lobata* root extracts.
