# Supplementary material for: Solvent-Dependent Chemical Profiles and Biological Activities of Pueraria lobata Root Extracts
Source: Molecules. 2026 Mar 13;31(6):965. doi: 10.3390/molecules31060965 (PMC13028639; doi:10.3390/molecules31060965)
Supplement: Supplementary file 1 [file molecules-31-00965-s001.zip › molecules-4145097-supplementary.pdf]

Supplementary Figure S1. Three-dimensional UHPLC–PDA spectral maps (200–400 nm) of different solvent extracts, illustrating the overall UV fingerprint profiles.

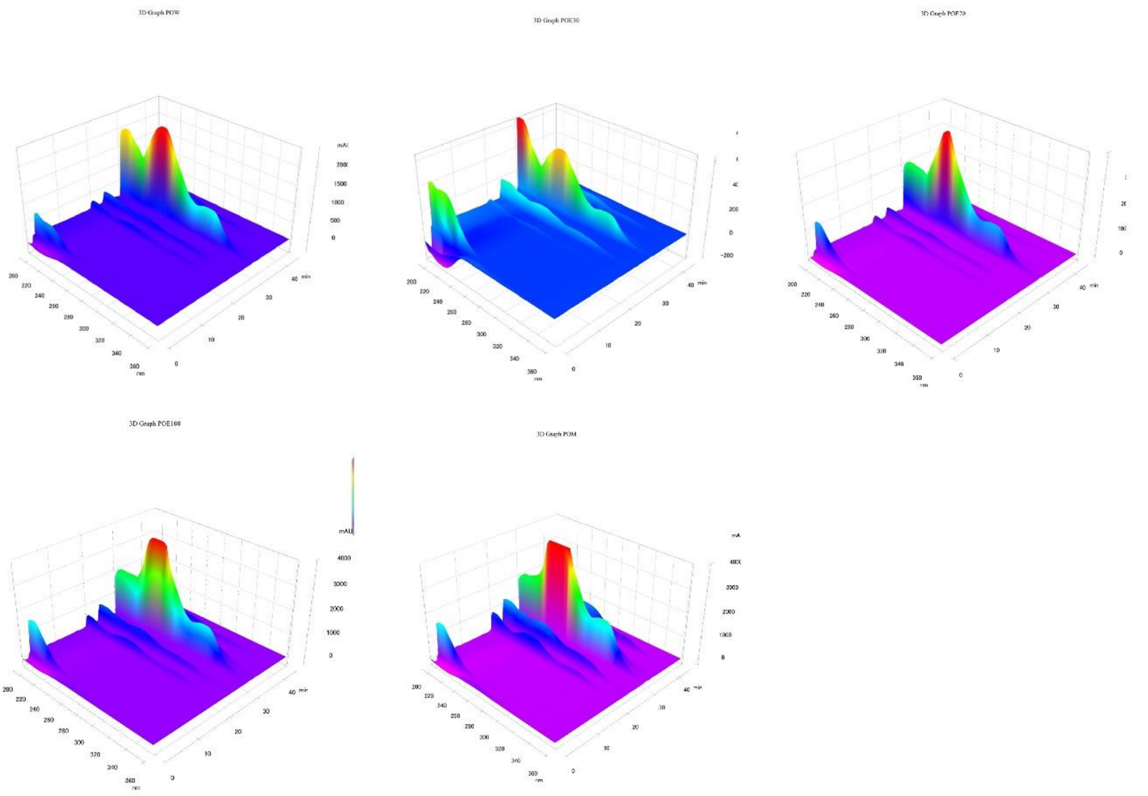

Supplementary Figure S2. UHPLC–PDA chromatogram (254 nm) of the reference standard puerarin. The standard peak of puerarin was detected at a retention time (RT) of approximately 31.0 min. Representative chromatographic peaks corresponding to puerarin were observed at similar retention times in the solvent extracts.

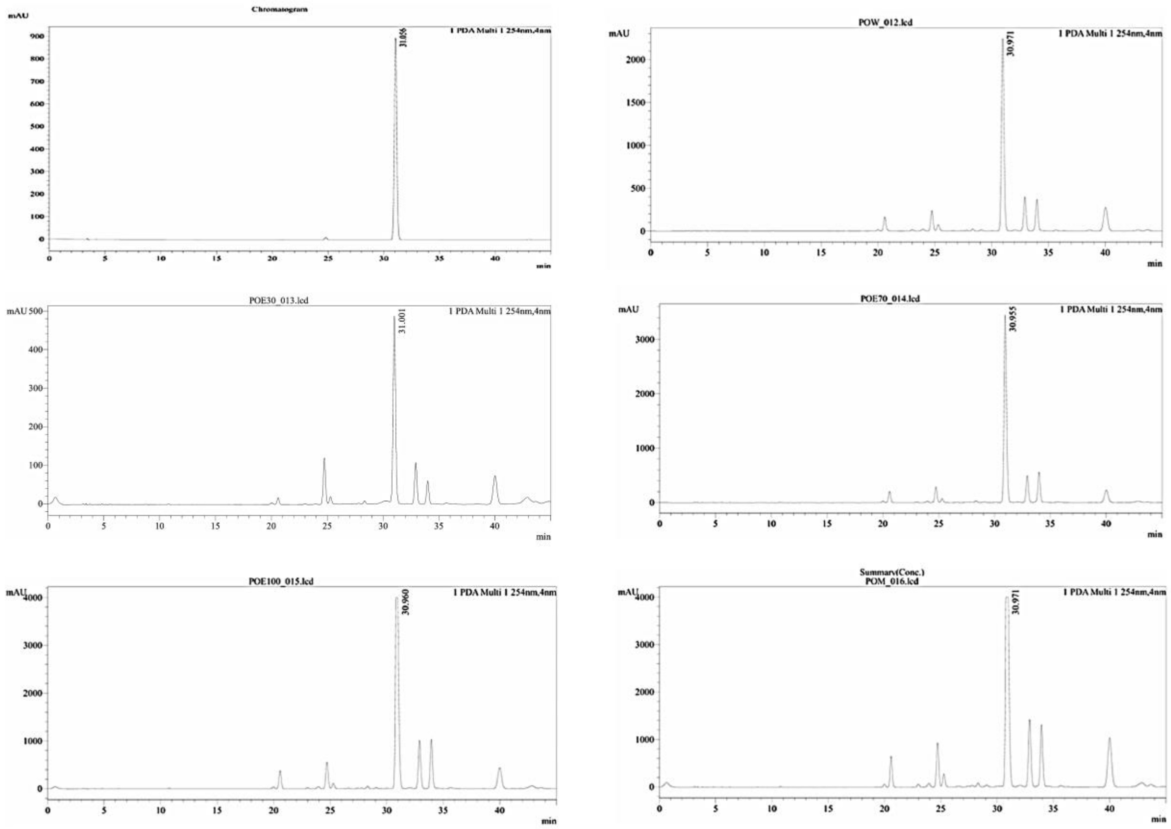

Supplementary Figure S3. UHPLC–PDA chromatogram (254 nm) of the reference standard daidzin. The standard peak of daidzin was detected at a retention time (RT) of approximately 40.0 min. Chromatographic peaks with comparable retention behavior were observed in the solvent extracts.

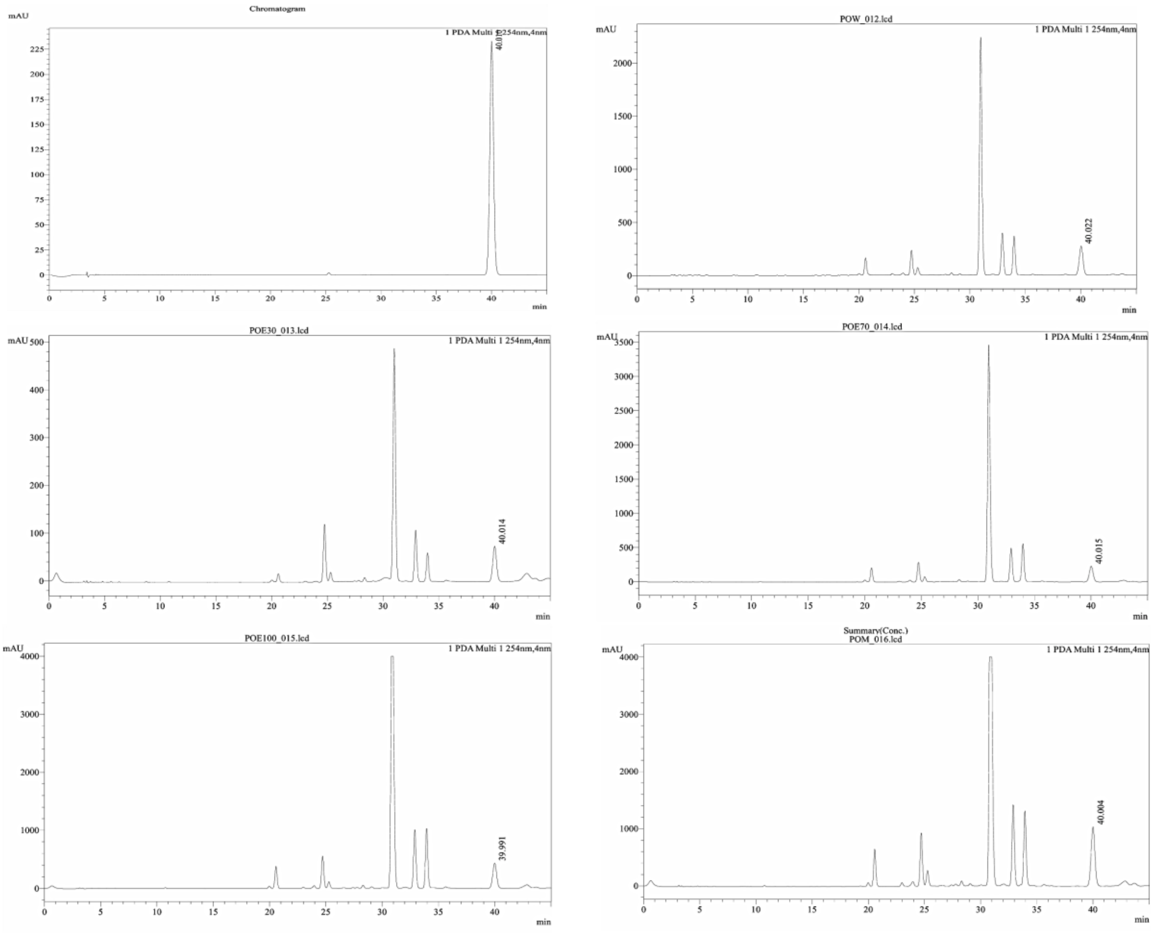

Supplementary Table S1. Compound component analysis of *P. lobata* root extracts.

Compound	Analytical method	Retention time (min)
Puerarin	UHPLC-PDA (254 nm)	~31.0
Daidzin	UHPLC-PDA (254 nm)	~40.0
